# The Role of the Redox Enzyme p66Shc in Biological Aging of the Lung

**DOI:** 10.14336/AD.2023.0715

**Published:** 2024-04-01

**Authors:** Claudia F. Garcia Castro, Claudio Nardiello, Stefan Hadzic, Baktybek Kojonazarov, Simone Kraut, Mareike Gierhardt, Julia Schäffer, Mariola Bednorz, Karin Quanz, Jacqueline Heger, Martina Korfei, Jochen Wilhelm, Matthias Hecker, Marek Bartkuhn, Stefan Arnhold, Andreas Guenther, Werner Seeger, Rainer Schulz, Norbert Weissmann, Natascha Sommer, Oleg Pak

**Affiliations:** ^1^Excellence Cluster Cardio-Pulmonary Institute (CPI), Universities of Giessen and Marburg Lung Center (UGMLC), Member of the German Center for Lung Research (DZL), Justus- Liebig University of Giessen, Giessen, Germany.; ^2^Instituto de Investigación en Biomedicina de Buenos Aires (IBioBA), CONICET - Partner Institute of the Max Planck Society, Buenos Aires, Argentina.; ^3^Max Planck Institute for Heart and Lung Research, Bad Nauheim, Germany.; ^4^Institute of Physiology, Justus-Liebig University of Giessen, Giessen, Germany.; ^5^Institute for Lung Health (ILH), Giessen, Germany.; ^6^Institute of Veterinary Anatomy, Histology and Embryology, Justus-Liebig University of Giessen, Giessen, Germany.; ^7^European IPF Registry & Biobank (eurIPFreg), Giessen, Germany.; ^8^Agaplesion Evangelisches Krankenhaus Mittelhessen, Giessen, Germany

**Keywords:** p66Shc, lung function, pulmonary vasculature, right ventricular function

## Abstract

The mitochondrial adaptor protein p66Shc has been suggested to control life span in mice via the release of hydrogen peroxide. However, the role of p66Shc in lung aging remains unsolved. Thus, we investigated the effects of *p66Shc^-/-^* on the aging of the lung and pulmonary circulation. *In vivo* lung and cardiac characteristics were investigated in *p66Shc^-/-^* and wild type (WT) mice at 3, 12, and 24 months of age by lung function measurements, micro-computed tomography (µCT), and echocardiography. Alveolar number and muscularization of small pulmonary arteries were measured by stereology and vascular morphometry, respectively. Protein and mRNA levels of senescent markers were measured by western blot and PCR, respectively. Lung function declined similarly in WT and *p66Shc^-/-^* mice during aging. However, µCT analyses and stereology showed slightly enhanced signs of aging-related parameters in *p66Shc^-/-^* mice, such as a decline of alveolar density. Accordingly, *p66Shc^-/-^* mice showed higher protein expression of the senescence marker p21 in lung homogenate compared to WT mice of the corresponding age. Pulmonary vascular remodeling was increased during aging, but aged *p66Shc^-/-^* mice showed similar muscularization of pulmonary vessels and hemodynamics like WT mice. In the heart, *p66Shc^-/-^* prevented the deterioration of right ventricular (RV) function but promoted the decline of left ventricular (LV) function during aging. *p66Shc^-/-^* affects the aging process of the lung and the heart differently. While *p66Shc^-/-^* slightly accelerates lung aging and deteriorates LV function in aged mice, it seems to exert protective effects on RV function during aging.

## INTRODUCTION

The proportion of the elderly population is growing around the world. Therefore, understanding the physiological changes associated with the aging process is crucial to meet the health needs of the elderly. The human lung partially grows due to neoalveolarization during childhood and adolescence and continues to mature fully by the age of 20-25 [1]. Lung function remains stable up to 35 years of age, and from this point on, it gradually deteriorates over the years. Structural alterations during the aging of the human lung are characterized by an increase in the size of alveoli without changing their number due to modifications of collagen fibers and alveolar duct dilation [2]. This enlargement of the alveolar space needs to be distinguished from emphysema, characterized by alveolar destruction. As a result of increased alveolar size, the surface tension is decreased, and thus the lung becomes more compliant. The loss of elastic recoil pressure, in combination with altered breathing mechanics, due to reduced respiratory muscle strength, results in decreased expiratory flow, increased residual volume, and functional residual capacity (FRC) [3].

Furthermore, with age, arterial oxygen tension declines, caused by diffusion limitation and ventilation/perfusion (V/Q) inequality [4]. The vascular alterations, including an increase in the loss of vessels (impaired angiogenesis), increased vascular stiffness of large arteries, and endothelial dysfunction, led to increased pulmonary arterial pressure (PAP) in the elderly, which promoted V/Q mismatch [5]. Additionally, age-related immune dysfunction may further contribute to enhanced susceptibility to various lung diseases, such as chronic obstructive pulmonary disease (COPD) [6].

Physiological aging is an intrinsic process that targets cell regeneration and repair. Different signaling pathways regulate it, leading to genomic instability, telomere attrition, epigenetic alterations, loss of proteostasis, deregulated nutrient sensing, cellular senescence, stem cell exhaustion, altered intracellular communications, and mitochondrial dysfunction under the influence of endogenous and exogenous factors [7]. Although the biological basis of aging is far from being understood, one central hypothesis suggests that aging is related to the accumulation of cellular damage caused by the release of reactive oxygen species (ROS) from mitochondria (“free radical theory”) [8] or redox modifications of proteins (“redox theory”) [9]. However, increased mitochondrial ROS and oxidative stress in genetically modified mice do not accelerate the aging process [10, 11]. Likewise, genetic manipulations that increase antioxidant defense mechanisms do not extend lifespan [12]. Furthermore, some evidence suggests that increased ROS can prolong lifespan in yeast and C. elegans [7]. Therefore, López-Otín’s landmark review on the hallmarks of aging suggests that, as individuals age chronologically, the ROS levels increase as a mechanism to maintain survival. However as aging progresses, ROS eventually aggravates the age-associated damage instead mitigating it [7]. Nevertheless, it is important to consider that the direct role of ROS in aging is still not fully understood. This is due to variations in individuals’ genomes and their unique response to the exposome, which encompasses cumulative environmental influences [9].

ROS can contribute to the aging process through various cellular pathways that are recognized as hallmarks of aging [8, 9] including induction of cellular senescence [13]. Senescence has a dual nature, as it prevents the propagation of stressed or damaged cells that may cause cancer, yet it also hampers tissue repair and regeneration by restricting the proliferation of stem or progenitor cells; additionally, the senescence-associated secretory phenotype (SASP) initially aids wound healing but, when persistent, can induce pathological inflammation, thus playing a role in diverse age-related conditions, including cancer [14]. Thus, while senescent cells have been implicated in supporting physiological functions, their accumulation in aging lungs can impair lung function and contribute to the exacerbation of lung diseases such as COPD, lung fibrosis, and even asthma [15].

The increase of intracellular ROS can lead to cellular senescence through the activation of p53 that triggers the expression of pro-senescence targets, including p21 [13]. Alternatively, dysfunctional mitochondria can trigger a ROS-JNK (C-Jun N-terminal kinase) retrograde signaling pathway that drives cytoplasmic chromatin fragment formation and subsequently the induction of the SASP [16].

The adaptor protein p66Shc has been postulated as a regulator of mitochondrial ROS [17-19] and thus p66Shc has been intensively investigated in the aging process. Multiple studies have demonstrated the correlation between cellular senescence and the upregulation of ROS in humans, specifically through the increased expression of p66Shc [20-22]. Low doses of hydrogen peroxide induced senescence in a hepatoblastoma cell line, and this effect was found to be absent in p66Shc knockdown [21]. Furthermore, p66Shc expression was associated with upregulated expression of the senescence markers including p16, p21, and p53 [21].

The p66Shc adaptor protein is the largest isoform encoded by the ShA locus of the human chromosome 1 or the mouse chromosome 3. The ShA locus encodes three different alternatively spliced adaptor proteins (p46Shc, p52Shc, and p66Shc). p46Shc and p52Shc are ubiquitously expressed and participate in the activation of Ras by receptor tyrosine kinases.

In contrast, p66Shc is specifically expressed in the heart, kidney, lungs, liver, and spleen and plays an essential role in the mitochondrial response to oxidative stress [23]. Under basal conditions, a large percentage of p66Shc protein is located in the cytoplasm, while just 10% is located in the mitochondrial compartment [24]. Upon p53 activation by different stress stimuli (oxidative stress, DNA damage, insulin receptor activation), p66Shc translocates to the mitochondria, where it binds cytochrome c and acts as an oxidoreductase, generating superoxide and hydrogen peroxide as a signaling molecule for apoptosis [17-19]. In addition to its role in the mitochondrial oxidative stress response, p66Shc increases the activity of nicotinamide adenine dinucleotide phosphate oxidases (NADPH oxidases) and suppresses the expression of antioxidants, including superoxide dismutase and glutathione peroxidase [25]. Therefore, it has been initially assumed that p66Shc controls lifespan in mice [26]. This hypothesis was supported by data showing that *p66Shc*^-/-^ live longer [23, 27]. Moreover, *p66Shc*^-/-^ mice are protected against different age-related diseases like atherosclerosis, diabetes-induced glomerulopathy, and Alzheimer’s disease [28-31]. However, Ramsey et al. did not find any differences in lifespan between wild type (WT) and *p66Shc*^-/-^ mice, suggesting that *p66Shc* deletion increases lifespan only under certain circumstances [32].

Thus, our hypothesis suggests that *p66Shc^-/-^* may provide protection against age-related alterations in the lung and pulmonary circulation by inhibiting p66Shc-mediated senescence. Therefore, we investigated the differential effects of *p66Shc*^-/-^ on the aging of the lung and pulmonary circulation.

## MATERIALS AND METHODS

### Human lung tissue samples

Human lung tissue samples were obtained from healthy donors. The studies were approved by the Ethics Committee of the Justus- Liebig- University School of Medicine (AZ 31/93, 10/06,220/18). Subjects were divided into young (20-30 years old), middle (40-50 years old) and old (60-80 years old) groups. The cohort included 6 females and 8 males. Characteristics of the participants are given in Supplementary Table 1.

### Animals and experimental design

All animal experiments were performed following the Directive 2010/63/EU of the European Parliament on the protection of animals used for scientific purposes and approved by local authorities (Regierungspräsidium, Giessen). *p66Shc*^-/-^ mice were bred and naturally aged in our central animal facility. C57BL/6J (WT) mice were bred in our central animal facility (3-month- old group) or purchased from Jackson Laboratories at eight months of age and naturally aged (12-24-month-old group) before experimentation. Mice of both sexes were randomly assigned and investigated at 3, 12, or 24 months. These time points correspond to different stages of human life: adults 20-30 years old (3-4 months in mice), middle-aged 38-47 years old (12-14 months in mice), and old 56 to 70 years old (20-24 months in mice). The approximate mouse/human comparison was based on previous reports [33]. All animals had housed four mice per cage at the temperature of 23±1°C under light-controlled conditions (inverse 12:12 hour light-dark cycle). Food and water were provided *ad libitum*. Due to animal availability during experiments, the number of animals in the group varied. In the WT groups, numbers of animals were as follows: n=10, n=12 and n=9 at the age of 3, 12 and 24 months, respectively. In the *p66Shc*^-/-^ groups, numbers of animals were as follows: n=10, n=6 and n=8 at the age of 3, 12 and 24 months, respectively. In all experiments, no differences were found between male and female mice (data not shown). All animals were used for determination of *in vivo* hemodynamic, echocardiography, lung function measurements and µCT imaging. Randomly assigned mice were chosen for stereology (n=5) and for histological analyses of pulmonary vasculature (n=6). N-numbers for *in vivo* lung function, hemodynamics, echocardiography and µCT may differ from initial n-numbers due to technical issues during measurement (e.g., dislocation of measurement catheter or position of microchip that was used to track mice). The *in vivo* lung function data (stat. compliance, inspiratory capacity, hysteresis and tissue elastance) with coefficient of determination (COD) greater than 0.95, were excluded from analysis.

### In vivo lung function

Initially, mice were anesthetized in a chamber using 3% isoflurane. During hemodynamic and lung function measurements, anesthesia was decreased to 1.5-2.0% isoflurane to ensure deep state of anaesthesia tailored to each individual [34, 35]. Lung function was measured with a FlexiVent system (Flexivent FX, Canada) as previously described [34]. Hysteresis was calculated as the surface area between the ascending and descending portions of the pressure-volume loop. After measurement of lung function, a Millar catheter was placed in the right ventricle via the right external jugular vein to assess the right ventricular systolic pressure (RVSP) as previously described [34, 35].

### Echocardiography

All echocardiographic studies were performed as previously described [35]. Briefly, mice were anesthetized with 3% isoflurane and placed on the heated stage of the Vevo 2100 (VisualSonics, Canada) under continuous anesthesia with 1.5-2.0% isoflurane. The heart rate was monitored during the entire study by electrocardiogram (ECG) recording. Tricuspid annular plane systolic excursion (TAPSE) and RV wall thickness (RVWT) were measured in the apical four-chamber view and in a modified parasternal short-axis view, respectively. LV ejection fraction (LVEF) was measured in the parasternal long-axis view. IVRT was corrected for heart rate, calculating the RR time interval and using the formula: IVRT/RR%. Cardiac output (CO) was calculated as the product of the velocity-time integral of the pulsed-Doppler tracing in the right ventricular outflow tract, the cross-sectional area of the right ventricular outflow tract, and the heart rate. Cardiac index (CI) was calculated by dividing the CO by body weight.

### In vivo micro-computed tomography (µCT) imaging

Images were acquired using a Quantum GX microCT scanner (PerkinElmer, USA). The mice were anesthetized by the inhalation of 3% isoflurane in oxygen. After that, mice were placed on a scanner platform with a nose cone, supplied with 1.5-2.0% isoflurane. The scanner platform was translated longitudinally to align the animal chest within the center of the field of view. The scanner’s complementary metal-oxide-semiconductor X-ray flat-panel detector was set to allow image acquisition with an X-ray tube voltage of 90 kV and current of 80 μA. For Hounsfield unit (HU) calibration, a phantom consisting of an air-filled 1.5 ml tube inside a water-filled 50 ml tube (water: 0 HU, air: -1000 HU) was scanned. µCT data were collected in list mode over a single complete gantry rotation with a total rotation time of 4 min (in total, 14688 frames were collected). After data collection, the mouse was removed from the scanner and monitored during recovery from anesthesia. Raw projection images were processed using a proprietary algorithm for intrinsic retrospective respiratory gating and then reconstructed using a filtered back-projection algorithm on a dedicated graphics processing unit. Reconstructed volumes were loaded and processed by Analyze 12 software (Analyze Direct, Mayo Clinic). Lung segmentation and quantitative analysis of the lung density, functional residual capacity (FRC) and lung’s air to tissue ratio were performed as described [36].

### Design-based stereology

The analysis of the lung architecture was performed following the recommendations of the American Thoracic Society/European Respiratory Society for quantitative analysis of lung structure [37]. Briefly, lungs were flushed blood-free with saline via the pulmonary artery. Afterward, the right lung lobe was clamped, removed, and stored in liquid nitrogen for further analyses. The left lobe was fixed with formaldehyde by intratracheal infusion of 4.5% formaldehyde at 15 cmH_2_O and via the pulmonary artery at 20 cmH_2_O for 15 min. The Archimedes principle assessed lung volume. Subsequently, the entire left lung was embedded in 3% agar and cut into ~3mm sections. The sections were dehydrated and embedded in paraffin next to each other. 2μm sections were prepared and stained with Verhoeff-van Giesson. The assessment of the number of alveoli was performed on two alternate sections with a thickness of 2μm at a distance of 2μm from each other, which were placed side by side on the same slide and examined using a light microscope (Leica, Germany) equipped with newCast software for stereology (Visiopharm, Denmark).

### Vascular morphometry

The degree of muscularization of pulmonary arterial vessels was determined in lung paraffin sections that were stained with the α-smooth muscle actin antibody (clone 1A4, dilution 1:900, Sigma-Aldrich, USA) to identify α-smooth muscle actin-positive cells and with the anti-mouse von Willebrand-factor antibody (GA527, dilution 1:900, Dako, Germany) to identify endothelial cells as in [35]. Vessels were categorized as fully muscularized (>70% vessel circumference α-smooth muscle actin positive), partially muscularized (5% to 70% vessel circumference α-smooth muscle actin positive), and non-muscularized (<5% vessel circumference α-smooth muscle actin positive).

### Primary cell isolation

Mouse primary alveolar pneumocytes type II (AECII), pulmonary artery smooth muscle cells (PASMC), and primary endothelial cells (EC) were isolated as previously described [38]. Mouse primary lung fibroblast (Fb) were isolated using the crawl-out technic as previously described [39].

### Electron spin resonance (ESR) microscopy

Intracellular and extracellular concentrations of ROS and reactive nitrogen species (RNS) were determined in lung homogenate by a Bruker electron spin resonance (ESR) spectrometer (EMXmicro; Bruker Biospin, Germany), using the spin probe 1-hydroxy-3-methoxycarbonyl-2,2,5,5-tetramethylpyrrolidine (CMH [40]). Lung tissue were homogenized in ESR-Krebs HEPES buffer (99.0 mM NaCl, 4.69 mM KCl, 2.5 mM CaCl_2_x 2H_2_O, 1.2 mM MgSO_4_ x 7H_2_O, 25 mM NaHCO_3_, 1.03 mM KH_2_PO_4_, 5.6 mM D(+) Glucose, 20 mM Na-HEPES, 25 μM deferoxamine, 5 μM diethyldithiocarbamate) containg protease inhibitor cocktail (Sigma-Aldrich, USA). Afterwards, samples were incubated for 30 min at 37°C with 0.5mM CMH. Following this procedure samples were collected into 1 ml syringes and flash frozen in liquid nitrogen. The X-Band (9.65 GHz) ESR measurements were performed at room temperature (20 - 22°C). The experimental parameters were as follows: G-factor 2.0063, center field ~3360 G, microwave power 2,000 mW, receiver gain 50 dB, time constant 10,24 ms, modulation amplitude 2,999 G, modulation frequency 100 GHz. The data from ESR microscopy were normalized to protein concentration measured by DC protein assay from Bio-Rad.

### Western blot

Lung tissue and cells were homogenized in lysis buffer (Cell signaling, USA). Samples were centrifuged at 14000g for 10 min at 4°C to remove debris. Protein concentration was determined using the DC protein assay from Bio-Rad. Samples for SDS-PAGE were denatured in a reducing Laemmli loading buffer at 99°C for 10 min. 25-30 µg of total protein per lane was electrophoretically separated in 12% TGX Stain-free Acrylamide gels and transferred into PVDF membranes (Pall Corporation, Germany) by a semidry-blotting method. The membranes were blocked using 6% non-fat milk in TBST buffer for 1 hour. Following, primary antibodies were used: anti-Shc (dilution 1:1000, 610879, BD Transduction Laboratories, USA), anti-p21 (dilution, 1:500, NBP2-29463, Novus Biologicals, USA), anti-LaminB1 (dilution 1:1000, #13435, Cell Signaling Technology, USA) and anti-β-actin (dilution 1:50000, A2228, Sigma-Aldrich, USA). Secondary antibodies were used: anti mouse (dilution 1:5000, W402B, Promega Madison, USA), and anti-rabbit (dilution 1:5000, W401B, Promega Madison, USA)

### Real-time RT-PCR

According to the manufacturer’s instructions, total mRNA was extracted from murine lung tissue using the RNeasy Mini kit (QIAGEN, Germany). RNA was reverse-transcribed using iScript cDNA Synthesis Kit (Bio-Rad, USA). qRT-PCR was performed in duplicates (technical replicates) with one µg of cDNA using the iQ SYBR Green Supermix (Bio-Rad, USA). Real-time PCR was performed with an Mx3000P (Stratagene, Germany) under the following condition: 1 cycle at 95°C for 10 min, then 40 cycles at 95 for 10 s, 59°C for 10 s, 72°C for 10 s. The Ct values were normalized to endogenous B2M (beta-2-microglobulin) expression.

### β -galactosidase staining

Senescence-associated β-galactosidase activity was measured in fresh lung samples. Lung cryosections of 8 µm were fixed with 4% PFA (Santa Cruz, USA) for 10 min and stained using the senescence β-galactosidase cell staining protocol (Cell signalling, USA) [41]. The counterstaining was performed using Nuclear Fast Red (Vector Laboratories, USA) following the manufacturer’s instructions. A549 cells and primary mouse fibroblast at late passage 6 were used as negative and positive controls respectively. Thirty different parenchymal fields were analyzed for each lung section. Positive cells were counted manually using the free ImageJ software.

### Statistical Analysis

Data were analyzed with one- or two-factorial linear models with R 4.3.0 (https://www.R-project.org). Where appropriate, the response data was log-transformed. Applied transformations are noted in the figure legends. The appropriateness of the assumptions was checked using residual diagnostic plots. For multi-group comparisons, post-hoc tests were performed using the function glht from the package multcomp 1.4-23 [42] and P-values were adjusted for multiple testing using the "single-step" method [43]. In two-factorial models with no relevant interaction, the main effects of the two predictors were tested by F-tests in corresponding linear models without interaction terms. P-values <0.05 were considered significant. Survival analysis was performed with GraphPad Prism 8.4 (GraphPad software, USA). Differences in survival were tested with a log-rank test. The numbers reported in all experiments refer to the numbers of individual mice or human subjects being analyzed. The sample size of all groups can be found in Supplementary Table 2.

## RESULTS

### Lung function was changed in WT and p66Shc^-/-^ to a similar extent during aging

Protein expression of p66Shc was detected in different primary lung cell types such as AECII, Fb, PASMC, and EC (Supplementary Fig. 1A). Accordingly, mRNA expression of p66Shc was detected in all lung cell population with some preference towards endothelial cells in humans [44]. To investigate whether p66Shc expression undergoes changes during aging in mice and humans, considering the differential regulation of aging between the two species [45], we measured its expression levels in lung homogenate of aged mice and humans. P66Shc levels were not significantly changed in human or mouse lung homogenate during aging (Supplementary Fig. 1B-C). Lung tissue from *p66Shc^-/-^* mice was used as negative control for the antibody (Supplementary Fig. 1D). Furthermore, we conducted an analysis of the data from the study by Schaum et al., which involved bulk RNA-sequencing of 17 organs and plasma proteomics across the lifespan of mice [46]. We analyzed these data to study the expression of p66Shc in 3-, 12- and 24-month-old mice in various organs. The bulk RNA-sequencing results indicated significant regulation only in gonadal fat and skin, demonstrating a decrease in p66Shc levels with age. (Supplementary Fig. 1E). These results support our data that do not indicate detectable alterations of p66Shc expression in the lung during aging.

**Figure 1.**
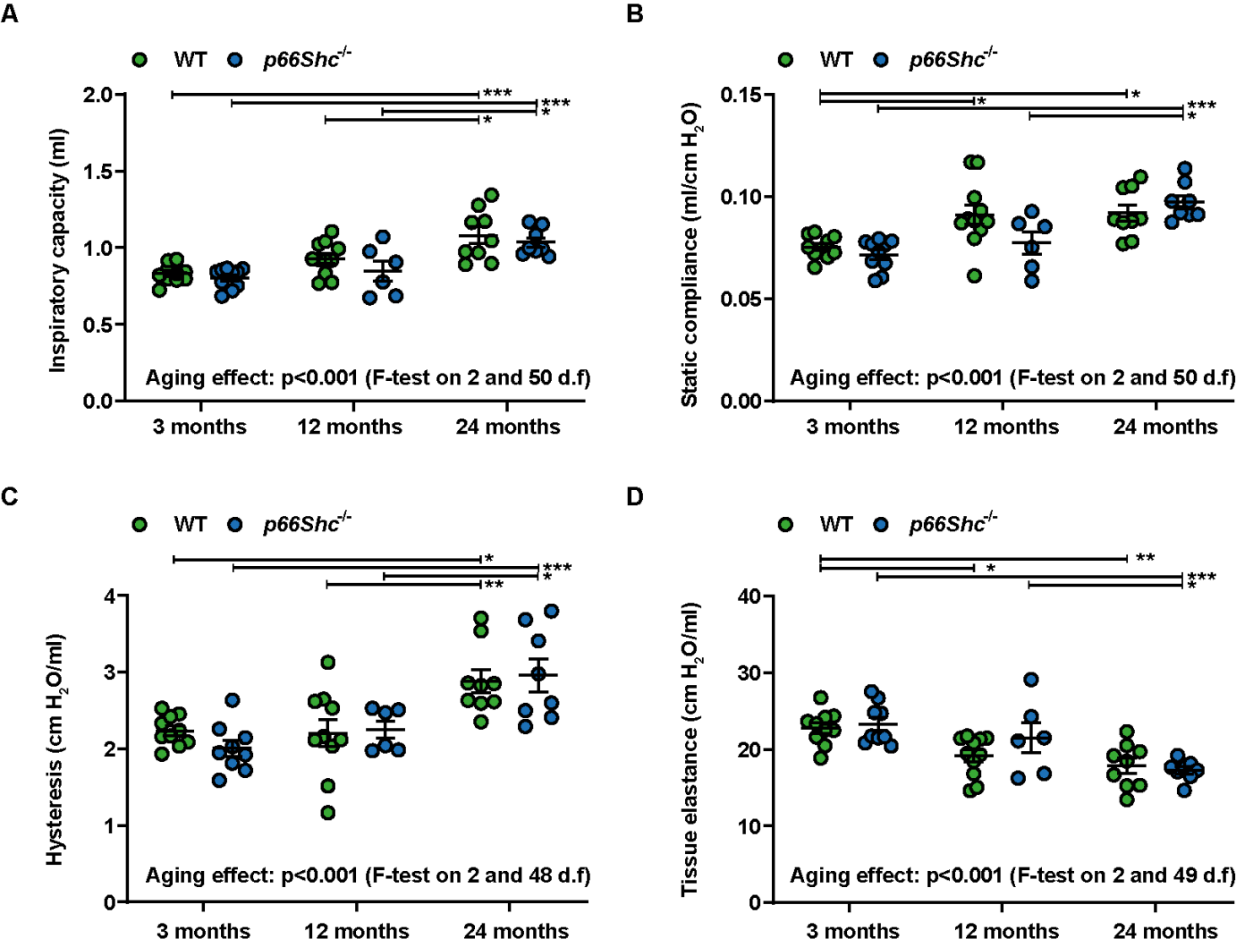
**Lung function changed in WT and *p66Shc*^-/-^ to a similar extent during aging**. *In vivo* lung function measurements for determination of (A) inspiratory lung capacity, **(B)** static compliance, **(C)** hysteresis, and (D) tissue elastance at different ages in WT and *p66Shc*^-/-^ mice using a Flexivent system. Multiple tests were performed as general linear hypothesis tests and *P*-values were adjusted for multiple testing using the "single-step" method. If there is no relevant interaction: main effects of the two predictors were tested by F-tests in corresponding linear models without interaction terms. n=6-11 mice per group. The sample size for each group is indicated in the Supplementary Table 2. *p <0.05, **p≤0.01, ***p ≤0.001.

Aging of the lungs in WT mice was characterized by an increase of inspiratory lung capacity (Fig. 1A), static compliance (Fig. 1B), and hysteresis (Fig. 1C), as well as by a decrease of tissue elastance (Fig. 1D). These data indicate an increased loss of lung tissue elastic recoil with aging, which leads to the decline of tissue elasticity (the ability to drive passive recoil), and ultimately increases lung compliance (distensibility of elastic tissue). Since hysteresis, the term used to describe the difference between inspiratory and expiratory compliance, is mainly affected by extracellular matrix composition and surfactant, it also increases with age. Age-related lung function alterations were similar in WT and *p66Shc^-/-^* mice (Fig. 1A-D).

### p66Shc^-/-^ accelerated lung aging in middle-aged mice

**Figure 2.**
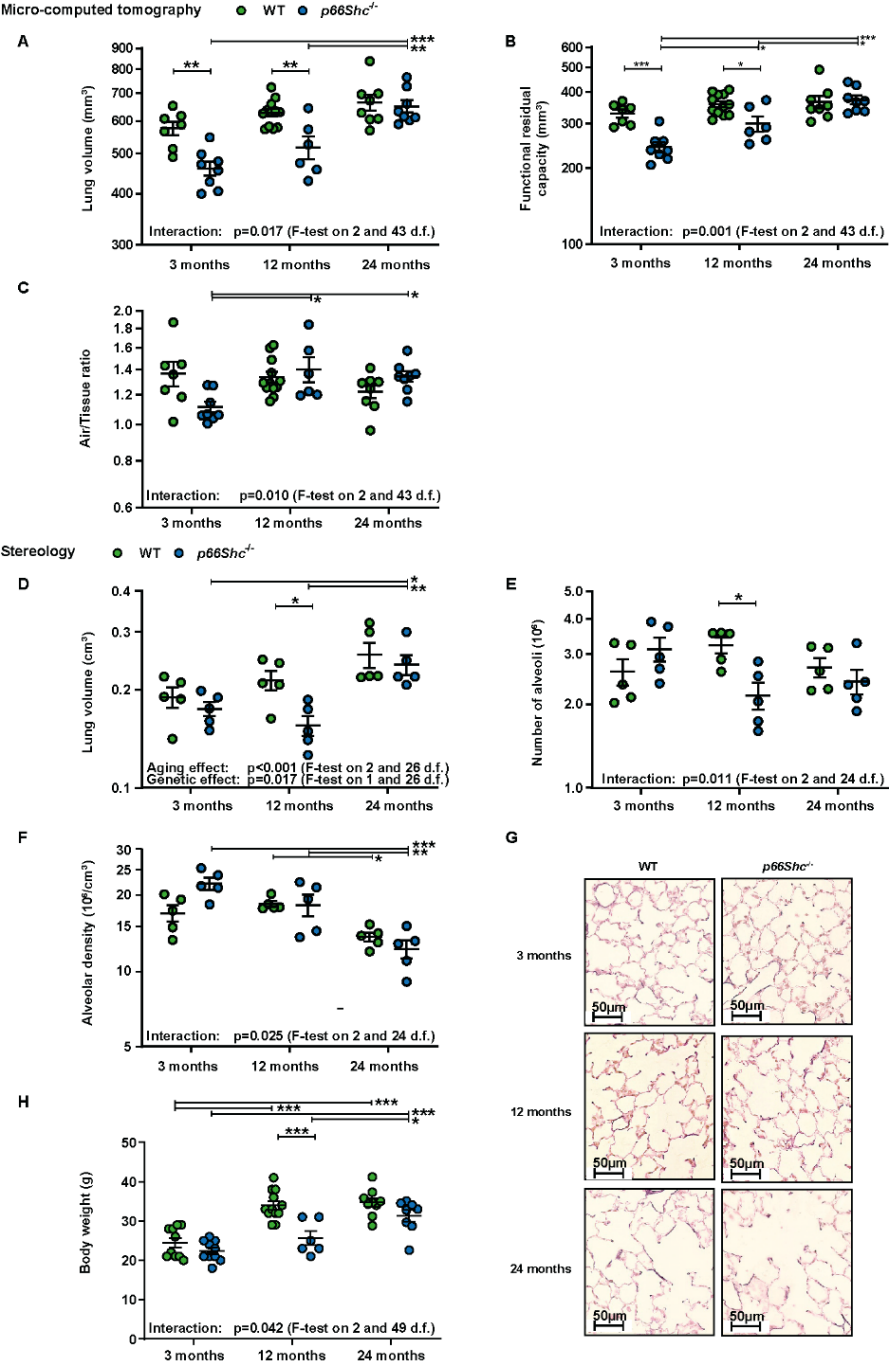
***p66Shc*^-/-^ accelerates lung aging in middle-aged mice**. **(A-C)** *In vivo* micro-computed tomography (µCT) for determination of (A) lung volume, **(B)** functional residual capacity, and (C) air/tissue ratio at different ages in WT and *p66Shc*^-/-^ mice. n=6-12 mice per group. **(D)** *Postmortem* analysis of lung volume. **(E-F)** Stereological analysis of (E) total number of alveoli and (F) alveolar density of the lungs’ left lobe at different ages in WT and *p66Shc*^-/-^ mice. n=5 mice per group. **(G)** Representative images of Verhoeff-van Gieson- stained 2 µm paraffin sections from WT and *p66Shc*^-/-^ mice at different ages. **(H)** Bodyweight of mice at different ages. n=6-12 mice per group. Data were log-transformed for analysis, except for body weight. Multiple tests were performed as general linear hypothesis tests and *P*-values were adjusted for multiple testing using the "single-step" method. If there is no relevant interaction: main effects of the two predictors were tested by F-tests in corresponding linear models without interaction terms. The sample size for each group is indicated in the Supplementary Table 2. *p<0.05, **p≤0.01, ***p ≤0.001.

For further in-depth investigation of lung function and structure, *in vivo* µCT measurements and postmortem stereological analyses were performed (Fig. 2). *In vivo* µCT demonstrated a more pronounced effect of aging in *p66Shc^-/-^* mice compared to WT mice, with a significant increase of lung volume (Fig. 2A), FRC (Fig. 2B) and air/tissue ratio in *p66Shc^-/-^* during aging (Fig. 2C). This effect was parallel by a lower lung volume and FRC at 3 and 12 months but not 24 months of age compared to the respective WT mice (Fig. 2A, B). Ex vivo measurement largely confirmed the µCT data (Fig. 2D-H), indicating an increase of lung volume during aging, albeit with different kinetics in the genotypes (Fig. 2D). Accordingly, the alveolar number was only decreased in *p66Shc^-/-^* mice at 12 months of age (Fig. 2E), while alveolar density decreased in both mouse strains (Fig. 2F, G). Statistical analysis indicated a more prominent decline in alveolar density and alveolar number in *p66Shc^-/-^* mice (Fig. 2E, F). Differences in lung volume and alveolar number at 12 months of age may have been related to a general effect of p66Shc on mouse growth, as the body weight and tibia length of *p66Shc^-/-^* was lower compared to WT mice at this age (Fig. 2H, Supplementary Fig. 2A). The survival analysis of WT and *p66Shc^-/-^* mice kept for 24 months did not reveal any significant effect of the genotype (Supplementary Fig. 2B). In summary, lung volume parameters and alveolar density decreased faster during aging in *p66Shc^-/-^* mice than WT mice. However, these effects were partially caused by differences at the baseline level of 3 months and may be affected by a general effect of *p66Shc^-/-^* on body growth.

**Figure 3.**
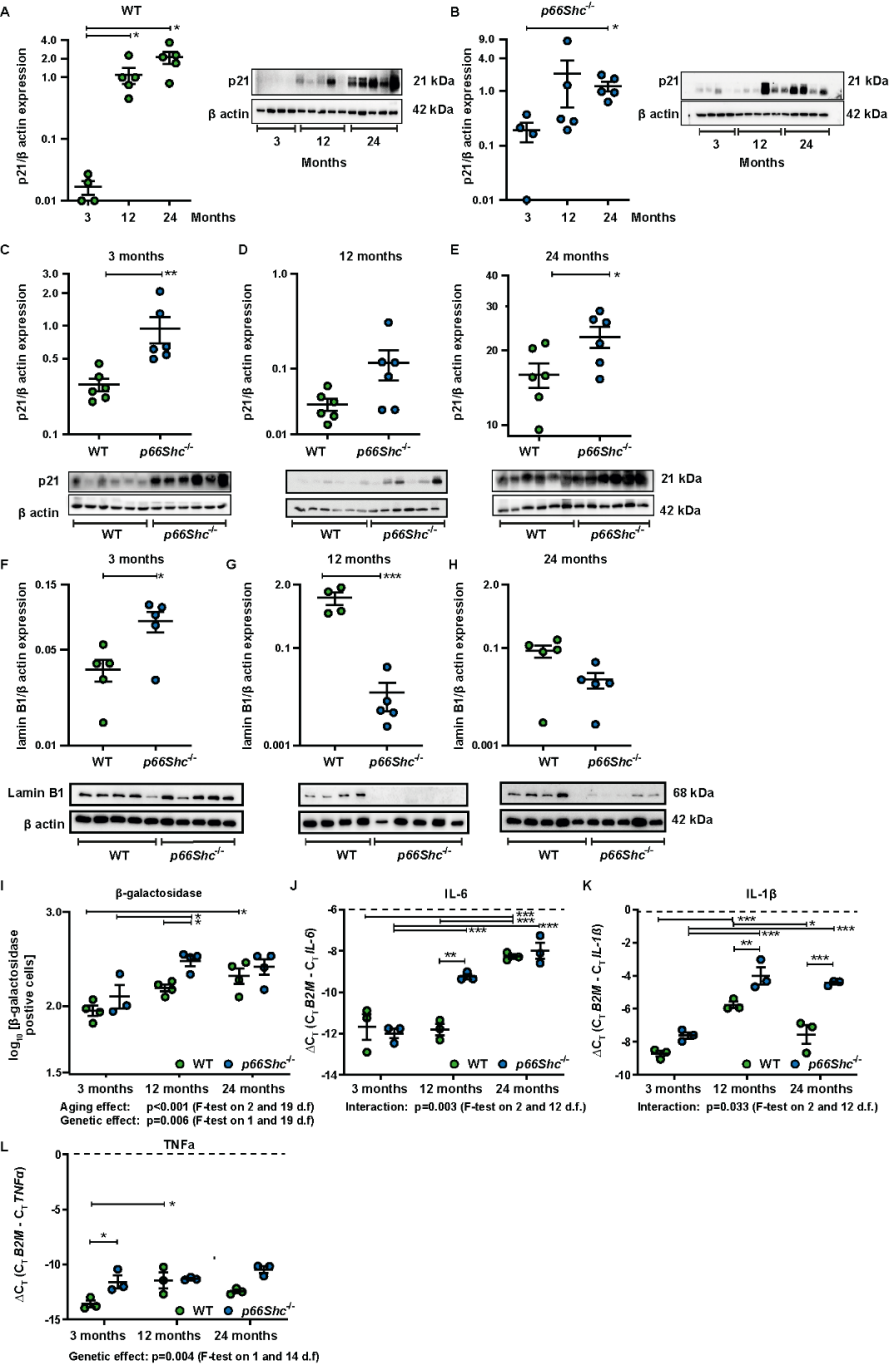
***p66Shc*^-/-^ induces premature cell cycle arrest during aging**. **(A-B)** p21 protein expression in lung homogenate at different ages in WT and *p66Shc*^-/-^ mice. n=4-5. **(C-E)** p21 protein expression in lung homogenates from WT and *p66Shc*^-/-^ mice of corresponding ages. n=6. **(F-H)** Lamin B1 protein expression in lung homogenates from WT and *p66Shc^-/-^* mice of corresponding ages. The expression of p21 and lamin B1 is depicted as a ratio relative to β-actin expression. **(I)** Senescence associated β-galactosidase (SA β-gal) activity in fresh cryo-sections from lungs of WT and *p66Shc*^-/-^ mice at 3, 12, and 24 months of age (*n*= 3-4 mice per group). Thirty parenchymal fields were randomly selected for each lung to quantify the number of β- galactosidase positive cells. **(J-L)** mRNA expression of (J) IL6, **(K)** IL-1β beta, and (L) TNFα in lung homogenates of *p66Shc*^-/-^ and WT during aging. n=3 mice per group. Data were log-transformed for analysis. **(A-B)** Tests were performed as general linear hypothesis tests and *P*-values were adjusted for multiple testing using the "single-step" method. **(C-H)** *P*-values are from Student’s. *t-tests.* (I-L) Multiple tests were performed as general linear hypothesis tests and *P*-values were adjusted for multiple testing using the "single-step" method. Main effects of the two predictors were tested by F-tests in corresponding linear models without interaction terms. n=3-6 mice per group. The sample size for each group is indicated in the Supplementary Table 2. *p <0.05, **p≤0.01, ***p ≤0.001.

### p66Shc^-/-^ induced premature cell cycle arrest during aging

To investigate if the decrease in the alveolar density in *p66Shc^-/-^* lungs was related to an increase of p66Shc-related cellular aging, we measured the senescent marker p21 in lung homogenates. First, we compared expression of p21, a cell cycle arrest marker during different ages, and found an increased expression during aging in both WT and *p66Shc^-/-^* mice (Fig. 3A, B). Afterwards, we directly compared p21 expression in WT and *p66Shc^-/-^* mice at specific ages (Fig. 3C-E). p21 expression was higher in lungs from 3 and 24 months old *p66Shc^-/-^* mice than WT mice (Fig. 3C-E). As further senescence marker, we investigated lamin B1 expression which is decreased during senescence [47]. Lamin B1 expression was higher at 3 months, but significantly lower at 12 months, and showed a tendency to decrease in 24-month-old *p66Shc^-/-^* lungs (Fig. 3F-H), thereby partially confirming the p21 results. Furthermore, we investigated the expression of senescence-associated β-galactosidase (Fig. 3I, Supplementary Fig. 3A). Consistent with p21 expression, we found a significant increase in β-galactosidase positive lung cells from both mouse strains during aging. Moreover, *p66Shc^-/-^* lungs from 12 months old mice had a higher level of β-galactosidase positive cells than WT mice of the same age (Fig. 3I). Metabolic changes and the adoption of a pro-inflammatory phenotype characterize senescent cells, known as the senescence-associated secretory phenotype (SASP) [48]. Interleukin-6 (IL-6) gene expression, one of the most prominent components of the SASP, increased during aging in WT mice, and this increase was more pronounced in *p66Shc^-/-^* mice at the age of 12 months compared to WT mice of the same age (Fig. 3J). Further markers of inflammation, which are also increased during senescence, IL-1β, and tumor necrosis factor α (TNFα), also increased during aging, and differences were observed between the genotypes (Fig. 3K, L).

**Figure 4.**
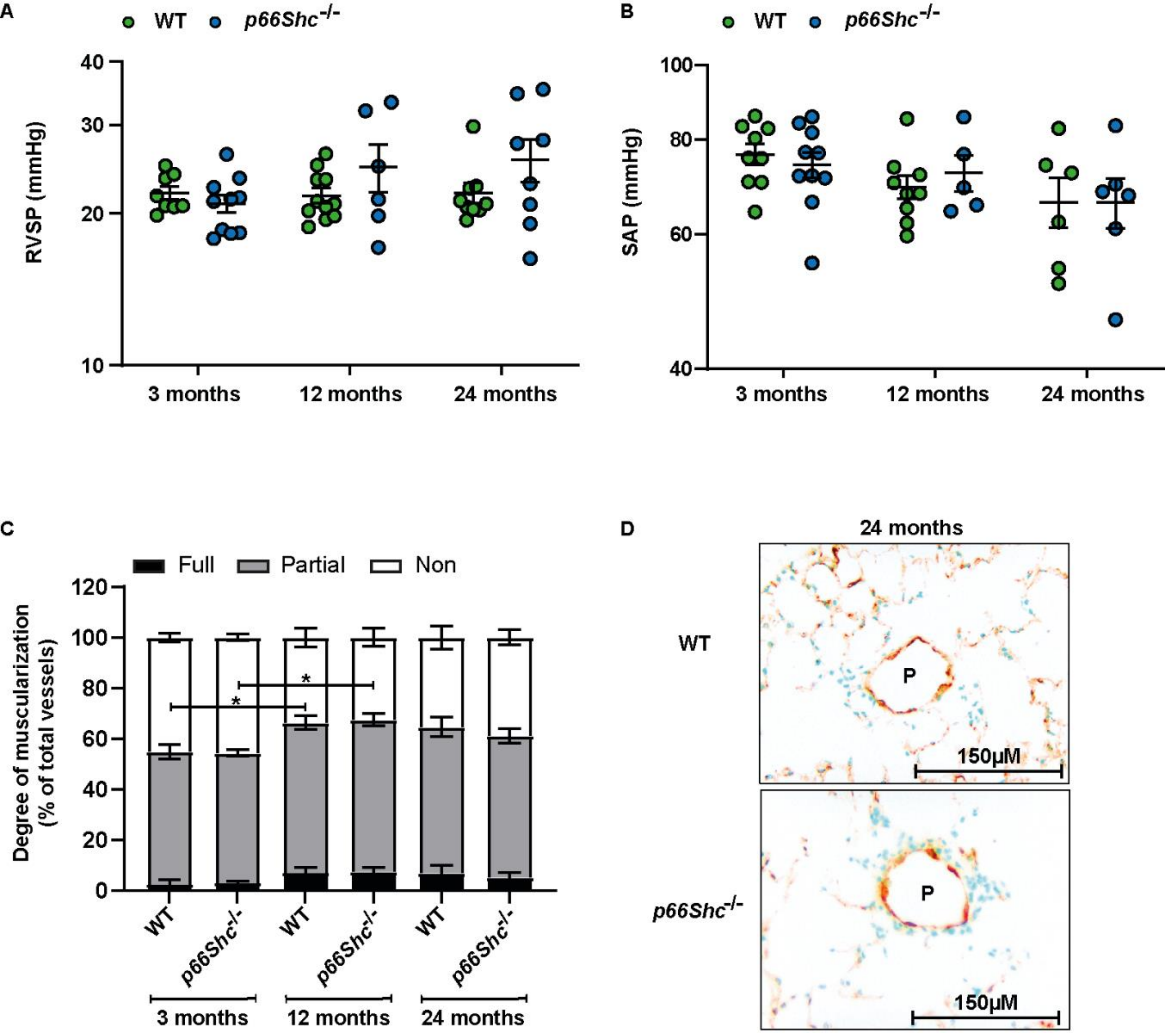
***p66Shc* ^-/-^ does not affect pulmonary vasculature during aging**. **(A-B)** *In vivo* hemodynamic measurement of RVSP and SAP at different ages in WT and *p66Shc*^-/-^ mice. n=5-12 mice per gorup. **(C)** The degree of muscularization of small vessels (20-100 µm) is given as the percentage of the number of all vessels (n=6 mice per group). Non - none muscularized, Partial - partially and Full - fully muscularized vessels. Representative images of lung paraffin slide double immunostained against α-smooth muscle actin (purple) and von Willebrand factor (brown) are provided. Data were log-transformed for analysis for (A-B). Multiple tests were performed as general linear hypothesis tests and *P*-values were adjusted for multiple testing using the "single-step" method. If there is no relevant interaction: main effects of the two predictors were tested by F-tests in corresponding linear models without interaction terms. The sample size for each group is indicated in the Supplementary Table 2. *p <0.05, **p≤0.01, ***p≤0.001.

**Figure 5.**
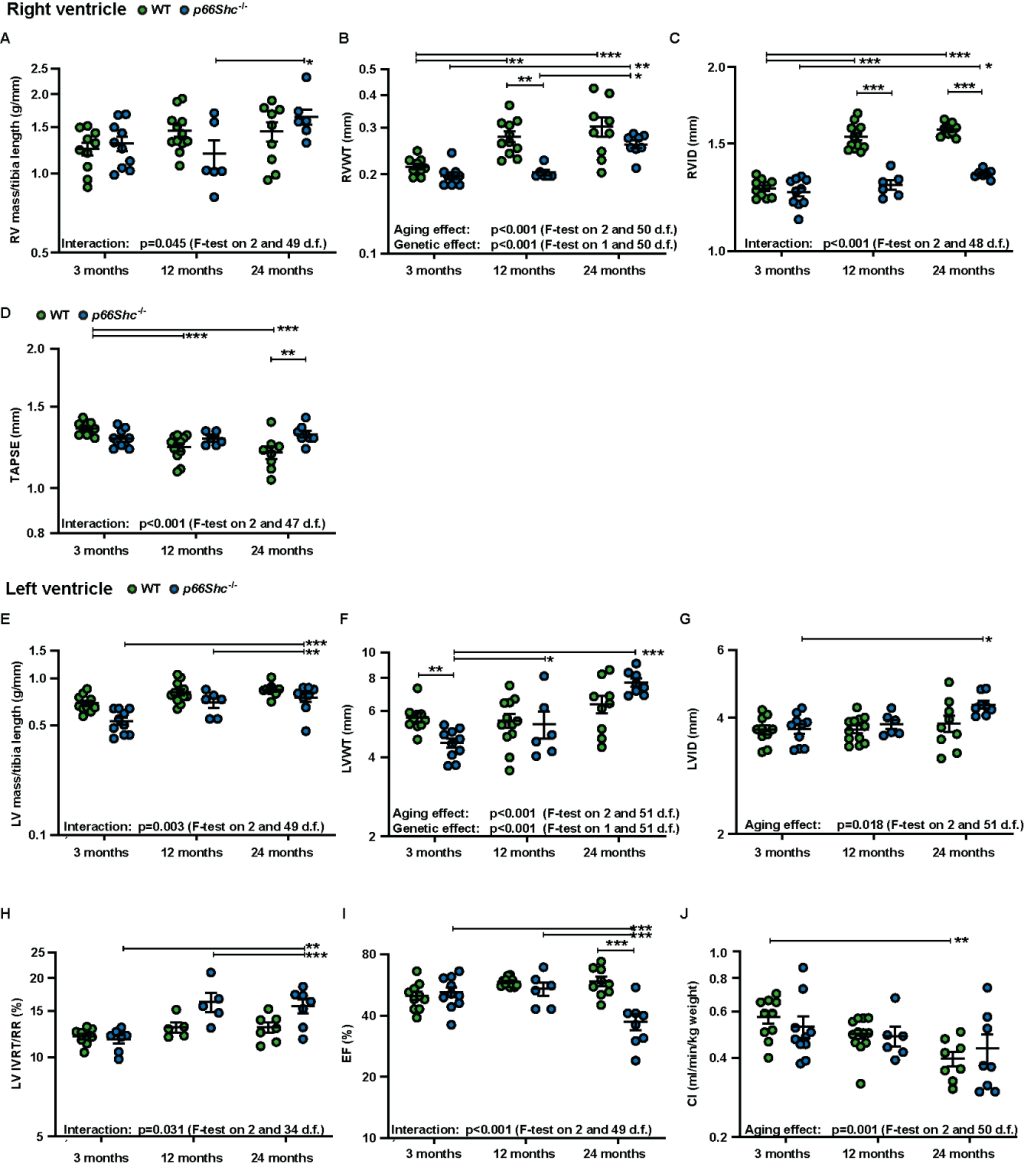
***p66Shc ^-/-^* differently affects the aging of the right and left ventricle**. **(A)** Ratios of the right ventricular (RV) mass to tibia length at different ages in WT and *p66Shc^-/-^* mice. **(B-D)** *In vivo* assessment of right heart characteristics by echocardiography at different ages in WT and *p66Shc^-/-^* mice for determination of (B) right ventricular wall thickness (RVWT), **(C)** right ventricular internal diameter (RVID), and (D) tricuspid annular plane systolic excursion (TAPSE). **(E)** Ratios of the left ventricular (LV) mass to the tibia length at different ages in WT and *p66Shc^-/-^* mice. **(F-J)** *In vivo* assessment of left heart characteristics by echocardiography at different ages in WT and *p66Shc^-/-^* mice for determination of (F) left ventricular wall thickness (LVWT), **(G)** left ventricular internal diameter (LVID), **(H)** isovolumic relaxation time corrected to heart rate (IVRT/RR), **(I)** ejection fraction (EF) and (J) cardiac index (CI). n=5-10 mice per group. Data were log-transformed for analysis. Multiple tests were performed as general linear hypothesis tests and P-values were adjusted for multiple testing using the "single-step" method. If there is no relevant interaction: main effects of the two predictors were tested by F-tests in corresponding linear models without interaction terms. The sample size for each group is indicated in the Supplementary Table 2. *p <0.05, **p≤0.01, ***p≤0.001.

Furthermore, we analyzed the levels of total extracellular and intracellular ROS/RNS in lung homogenates from both WT and *p66Shc^-/-^* mice during the aging process as increased sensescence can be regulated by ROS. This analysis was performed by the gold-standard method ESR microscopy using the CMH spin probe. Although ROS/RNS levels increased with aging, we did not detect a significant effect of the genotype (Supplementary Fig. 3B).

### p66Shc^-/-^ attenuated age-dependent effects in the right ventricle without significantly affecting the pulmonary vasculature, but accelerated age dependent effects in the left ventricle

The RVSP as well as SAP were similar in both mouse strains and did not significantly change during aging (Fig. 4A-B). However, morphometric analysis of the degree of muscularized vessels showed substantial remodeling in both genotypes at 12 months of age (Fig. 4C, D). Furthermore, aging increased RV hypertrophy determined by the ratio of the RV mass/tibia length, and the increase showed different kinetics in WT and *p66Shc*^-/-^ mice (Fig. 5A). Moreover, echocardiographic analysis revealed an increase in RV wall thickness, RV dilatation, and a decrease in TAPSE during aging (Fig. 5B-D). However, RV dilatation and reduction of TAPSE during aging were attenuated in *p66Shc*^-/-^ mice (Fig. 5C, D). Similar to the RV, the LV mass/tibia length, LV wall thickness, and LV dilatation increased during aging (Fig. 5E-G) with a more pronounced effect of LV mass/tibia length in *p66Shc*^-/-^ mice although LV wall thickness (Fig. 5E) was generally lower in *p66Shc*^-/-^ mice (Fig. 5F). Most importantly, LV function measured by isovolumetric relaxation time (IVRT; diastolic function) and ejection fraction (EF; systolic function) deteriorated during aging significantly in *p66Shc^-/-^* mice but not WT mice (Fig. 5H, I). However, although CI also decreased during aging, CI was not different between the genotypes (Fig. 5J).

## DISCUSSION

This study investigated for the first time the effects of *p66Shc^-/-^* on age-related functional and structural changes of the lung and pulmonary circulation in mice and described tissue-specific effects of *p66Shc-/-*. Following the initially proposed concept that *p66Shc^-/-^* exerts beneficial effects on aging-related alterations, our data show that *p66Shc^-/-^* protects against an age- induced decline in RV function. However, we found that p66Shc deletion does not attenuate age-related alterations of pulmonary function and/or structure. Most importantly, *p66Shc^-/-^* promoted decline in LV function.

Our data on the lack of a protective effect of *p66Shc*^-/-^ on lung aging is in accordance with previous investigations that did not detect differences between the lifespan of WT and *p66Shc*^-/-^ mice. These investigations showed that the lifespan of *p66Shc*^-/-^ mice was even decreased compared to WT mice when housed in a natural condition (outdoor) [49], in contrast to initial investigations that reported an increased lifespan of 30% in *p66Shc*^-/-^ mice [26]. In our studies, *p66Shc*^-/-^ mice also showed signs of accelerated lung aging, although it has to be taken into account that they started from different baseline levels.

The reasons for the changes observed specifically at 12 months in p66Shc and the absence of further progression at 24 months remain unknown. One possibility is that the decline in lung function and structure in *p66Shc^-/-^* mice reaches a plateau at 24 months. Interestingly, a study conducted by Schulte et al. revealed that greatest changes in murine lung function were observed during the early stages of adulthood, specifically between 3 months and 6-12 months [50]. This observation may provide an explanation for the accelerated effect of p66Shc deletion at this time point, as these mice lack "physiological" ROS [7] or a ROS-independent p66Shc-mediated function [51, 52]. It is worth noting that our study did not observe a difference in ROS production between WT and *p66Shc^-/-^* mice. However, it is plausible to consider that alterations in subcellular compartments, specific to certain cell types, stimuli-mediated responses, or subtle changes in ROS may play a crucial role in normal lung development, an aspect that could be lacking in *p66Shc^-/-^* mice. Additionally, it's important to note that we did not investigate mice beyond 24 months, so we cannot exclude the possibility of further deterioration in lung function/structure in *p66Shc^-/-^*mice.

Thus, *p66Shc*^-/-^ may have beneficial effects on the lung during embryonic development or at very early stages of life, which get lost during aging. Furthermore, we detected lower body weight in *p66Shc*^-/-^ mice, which may have contributed to lower lung volume and thus alveolar numbers (but not alveolar density). Decreased weight of *p66Shc*^-/-^ mice in the current study was similar to data from Berniakovich *et al*. [53] and Ramsey *et al*. [32] and may be caused by differences in the composition of the gut microbiota and their response to diet [54]. This finding promotes the earlier observation that the effect of *p66Shc*^-/-^ was affected by housing conditions, suggesting an interaction with environmental stress and the immune response. Previously, the impact of *p66Shc*^-/-^on the immune system with dysregulation of lymphocyte activation, survival, and apoptosis has been described [55]. This effect may be prominent in the lung, the organ with the most extensive interface with the environment and high accumulation of immune cells. We thus speculated that *p66Shc*^-/-^ might promote pulmonary inflammation and senescence - in contrast to the original concept of decreased ROS and subsequently decreased senescence and investigated markers of inflammation and senescence in lung homogenate. In contrast to previous observations on p66Shc and cellular senescence in the liver [21], we found enhanced senescence markers in the lungs of *p66Shc*^-/-^ by determination of p21, lamin B1 expression and β-galactosidase in the lung at 12 and/or 24 months of age. However, the last marker needs to be interpreted with caution since the expression of β-galactosidase in macrophages is a sign of polarization rather than senescence [56] and also may indicate effects of *p66Shc*^-/-^ on macrophage function. Interestingly, we discovered that while both senescence markers, p21 and lamin B1, indicate increased senescence in older *p66Shc*^-/-^ mice compared to WT mice, they demonstrated distinct regulation patterns in 3-month-old mice. Lamin B1 is known to regulate various cellular processes, including tissue development, cell cycle progression, cellular proliferation, and DNA damage response and thus may be differently regulated than p21 [57]. The underlying nature of this discrepancy warrants further investigation and should be addressed in future studies but may be related to our functional studies, which show differences at 3 months. The cellular mechanisms underlying the increase of p21 expression in *p66Shc*^-/-^ mice are not clear. Interestingly, recent publications suggest that p66Shc silencing could initiate a metabolic shift towards anaerobic glycolysis [58], which is known to up-regulate AMPK-p53 signaling pathways [59]. However, we cannot exclude an increase of ROS-p53-independent activation of p21 expression [60] in *p66Shc*^-/-^. Increased inflammation in *p66Shc*^-/-^ mice was shown by enhanced IL6, IL-1β, and TNFα mRNA levels, which are also essential components of the SASP and may indicate increased senescence [48, 61, 62]. It is important to note that the increased inflammation in *p66Shc^-/-^* mice may not only be a consequence of senescence but could also serve as a trigger for senescence [63]. Accordingly, the chronic exposure of *p66Shc*^-/-^ mice to cigarette smoke increased inflammation and accumulation of macrophages within and around bronchioles [64]. Thus, the effects of *p66Shc*^-/-^ on the immune system may overlap with its protective anti-oxidative effects and accelerate age-dependent pulmonary processes. However, it is important to interpret these findings with caution as the protein levels of IL6, IL-1β, and TNFα were not investigated in this study. Therefore, the specific impact of *p66Shc^-/-^* on the release of these cytokines cannot be definitively determined. However, there is a report that provides evidence for comparable levels of mRNA cytokine expression and cytokine release [65]. Additionally, it is worth considering that a systemic inflammatory response or organ dysfunction, which may contribute to the observed pulmonary effects, cannot be ruled out, as a global *p66Shc^-/-^* model was utilized in this study.

Furthermore, our findings are consistent with previous research [46], as we observed no significant differences in p66Shc protein expression in lung homogenates of mice or men during natural aging. In addition, our data revealed that p66Shc was expressed in all primary mouse cell lines investigated, including AECII, mFb, PASMC, and EC. These results align with the findings of Habermann et al. [44] in human data, where p66Shc was found to be expressed in the majority of lung cells, with a slight preference towards endothelial cells, as supported by The Human Protein Atlas (www.proteinatlas.org/). Therefore, it is plausible that p66Shc exerts cell-type-specific effects on lung function. Although we did not investigate posttranslational modifications of p66Shc, which may play a role in lung aging, p66Shc is mainly regulated by its Ser36 phosphorylation and, therefore, by mitochondrial translocation [66].

Considering the role of p66Shc for release of ROS [17-19, 25, 67], as well as the known association between ROS and senescence [13], we investigated the levels of ROS/RNS during aging by the gold standard method ESR microscopy using the spin probe CMH. Thereby, we found that ROS/RNS levels were increased in lung homogenate during aging. However, in contrast to previous findings [51, 67], we did not detect significant differences in the genotypes. This discrepancy could be explained by the fact that we measured ROS/RNS levels under basal conditions, while the effects of p66Shc can only be detected after stimulation. For instance, in our previous publication, we found that the level of hydrogen peroxide was not altered at the basal level in isolated mitochondria from *p66Shc*^-/-^ mice, but after rotenone stimulation [68]. Additionally, as mentioned earlier, it is important to consider the possibility that subcellular compartments or cell-specific ROS, which were not investigated in this study, might be absent in *p66Shc^-/-^* mice. Furthermore, p66Shc knockdown may affect senescence by its ROS-independent signaling [51, 52]. For instance, p66Shc can form a complex with forkhead box O3 (FOXO3a) and Pak-interacting exchange factor (β1Pix), facilitating β1Pix-induced FOXO3a phosphorylation [51]. Additionally, p66Shc can interact with focal adhesion kinase (FAK), leading to YAP/TAZ (Yes-associated protein; TAZ: Transcriptional coactivator with PDZ-binding motif) nuclear translocation and cell proliferation [52]. Both the FOXO3a and YAP/TAZ pathways are involved in the process of senescence [69, 70].

At the lung functional and structural level, our study showed that natural lung aging in WT mice was characterized by an increase in inspiratory lung capacity, FRC, lung compliance, and, accordingly, a decrease in tissue elasticity. These data are consistent with the observation that aging decreases lung tissue elastic recoil, which can cause the observed changes in lung functions and structure [71]. However, the number of alveoli did not change. Our findings differ from a study by Schulte et al., [50] which found similar lung functional/structural changes in WT mice during aging and a decrease in the number of alveoli at 12 months of age. Instead, our findings are similar to human lung aging. A reduction of elastic recoil causes increased lung compliance and FRC [71, 72], and there is an increase in the size of alveoli without changing their number [73]. In humans, the term “senile emphysema” has been used to describe these age-related morphological changes in the lung. It has to be pointed out that this is a misleading term as aging does not result in alveolar wall destruction and inflammation as in cigarette smoking-related emphysema [74]. Thus, our findings in WT mice resemble human airway and alveolar alterations during aging.

Going beyond previous studies on these pulmonary alterations, we also investigated the effect of aging on pulmonary circulation. Aging in both mouse strains did not significantly affect RVSP, but increased pulmonary vascular muscularization. Moreover, aging caused RV remodeling and decreased RV function. The effects in the RV were attenuated in *p66Shc*^-/-^ mice. The different effects of *p66Shc*^-/-^ on pulmonary vascular remodeling and the RV may be explained by a differential role of ROS in these processes, as described previously [75]. Only a few reports describe the effect of aging on the RV in experimental animals. A recent study in rats showed similar aging effects on the RVWT as our study [76]. Moreover, human studies report a decrease in RV function with age [77]. Thus, our data on RV remodeling and function in WT mice are in accordance with other studies. Moreover, a previous investigation did not show any differences in RV function between WT and p*66Shc*^-/-^ mice at baseline level or after pulmonary arterial banding [68], following our findings in 3 months old mice. In contrast to the effects on the RV, *p66Shc*^-/-^ accelerated LV aging. Accordingly, Hirschhäuser et al. showed that *p66Shc*^-/-^ changed the geometry of the LV and decreased LV fractional shortening at a basal level [68]. Our study has not addressed the cause of the different effects of *p66Shc*^-/-^ on RV and LV during aging. However, different signaling pathways in the RV and LV have been previously described [78].

### Conclusions

In our study, *p66Shc*^-/-^ resulted in organ-specific effects on the lung and the heart during aging. In contrast to our initial hypothesis that *p66Shc*^-/-^ may attenuate lung aging, *p66Shc^-/-^* showed signs of accelerated age-related structural pulmonary alterations which may be caused at least in part by promoting senescence. In contrast, *p66Shc*^-/-^ attenuated age-related RV remodeling and dysfunction while it exacerbated LV dysfunction. To gain a comprehensive understanding of systemic and organ-specific effects of *p66Shc^-/-^* during aging, further investigations in cell-type specific knockout mice (e.g., immune cell or endothelial specific knockout) are warranted. In particular, the cell-type specific effect of *p66Shc^-/-^* on ROS-dependent and -independent senescent pathways and SASP should be further investigated.

## Supplementary Materials

The Supplementary data can be found online at: www.aginganddisease.org/EN/10.14336/AD.2023.0715.


