# Supplementary material for: The Role of the Redox Enzyme p66Shc in Biological Aging of the Lung
Source: Aging Dis. 2024 Apr 1;15(2):911–26. doi: 10.14336/AD.2023.0715 (PMC10917546; doi:10.14336/AD.2023.0715)
Supplement: Supplementary file 1 [file AD-15-2-911-s.pdf]

SUPPLEMENTARY DATA

The Role of the Redox Enzyme p66Shc in Biological Aging of the Lung

Claudia F. Garcia Castro, Claudio Nardiello, Stefan Hadzic, Baktybek Kojonazarov, Simone Kraut, Mareike Gierhardt, Julia Schäffer, Mariola Bednorz, Karin Quanz, Jacqueline Heger, Martina Korfei, Jochen Wilhelm, Matthias Hecker, Marek Bartkuhn, Stefan Arnhold, Andreas Guenther, Werner Seeger, Rainer Schulz, Norbert Weissmann, Natascha Sommer, Oleg Pak

© 2023. Garcia Castro CF et al. Published online at http://www.aginganddisease.org/EN/10.14336/AD.2023.0715

SUPPLEMENTARY DATA

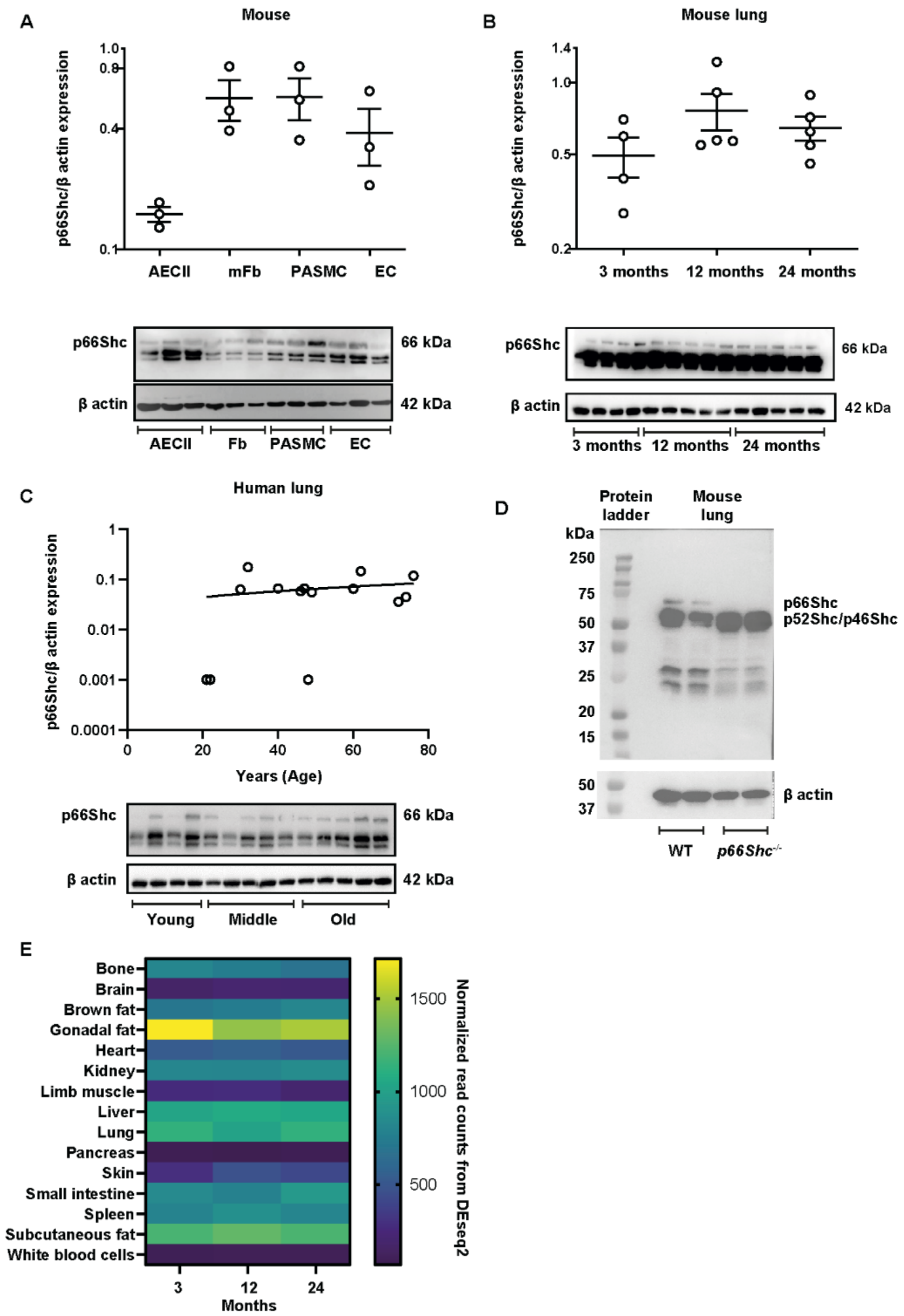

Supplementary Figure 1. p66Shc protein expression in different primary lung cells and during lung aging. (A) p66Shc protein expression in different primary mouse lung cell types. AECII- alveolar epithelial type II, FB- mouse fibroblast, PASMC- pulmonary arterial smooth muscle cells, and EC- endothelial cells. n=3 mice per group. (B-C) p66Shc protein expression in mouse (B) and human (C) lung homogenates at different ages. Mice were 3, 12, 24 months old and human samples were from young (20-30 years old), middle (40-50 years old) and old (60-80 years old) age groups. n=4-5 mice or humans per group. The expression of p66Shc is depicted as a ratio in relation to β-actin expression. (D) The specificity of AB against p66Shc was proven by using a negative control – lung tissue extracted from *p66Shc*$^{-/-}$ mice. (E) Expression of p66Shc mRNA in various organs during aging analyzed by data from GEO (GSE132040) [46]. Data were log-transformed for analysis. A-B) Tests were performed as general linear hypothesis tests and P-values were adjusted for multiple testing using the "single-step" method. C) Nonlinear regression analysis was performed with GraphPad Prism 8.4 (GraphPad software, USA). E) The expression of p66Shc mRNA in various organs was analyzed using one-way ANOVA with a Tukey posthoc test. The sample size for each group is indicated in the Supplementary Table 2.

© 2023. Garcia Castro CF et al. Published online at http://www.aginganddisease.org/EN/10.14336/AD.2023.0715

SUPPLEMENTARY DATA

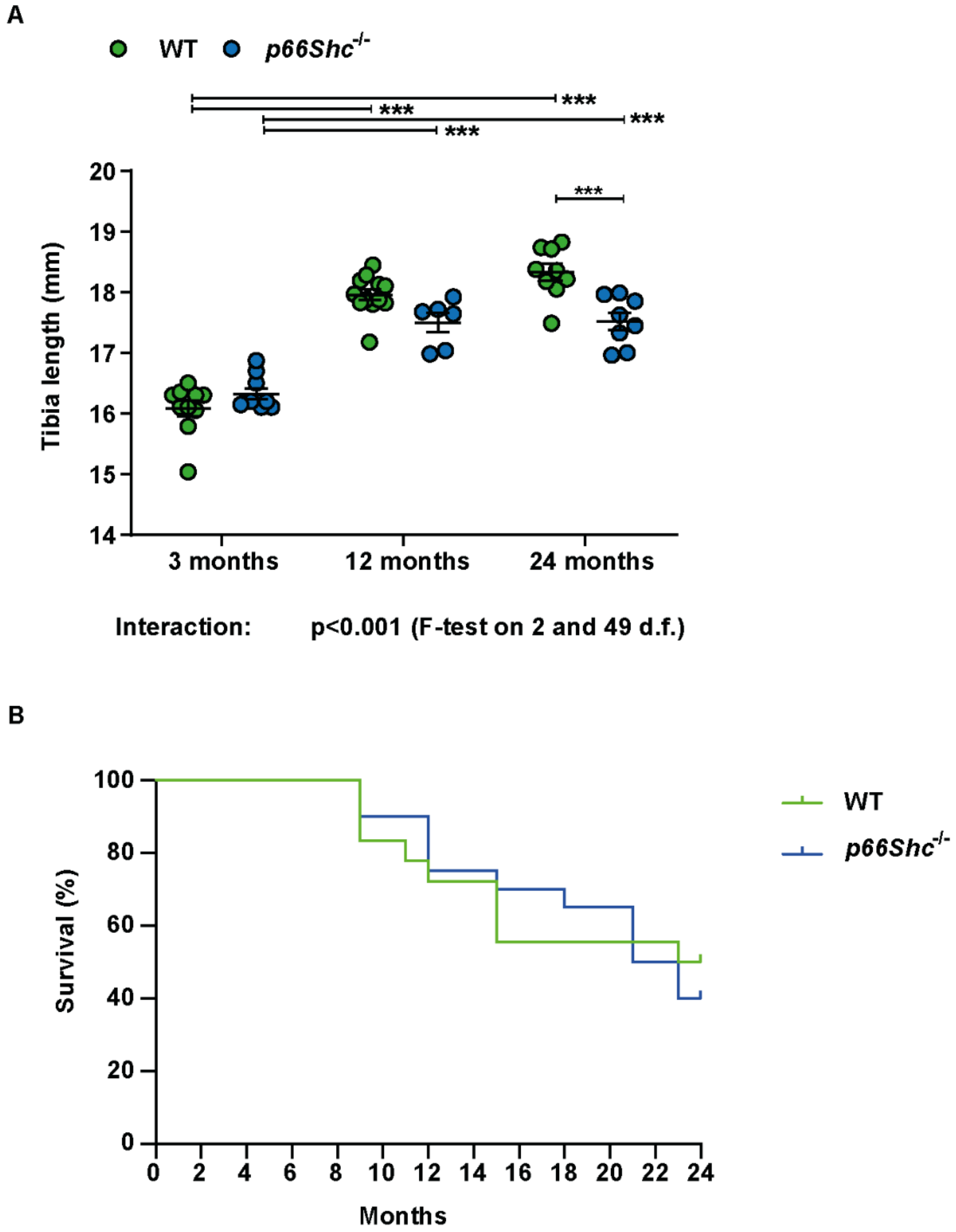

Supplementary Figure 2. The effect of *p66Shc* $^{-/-}$ on tibia length, and survival analysis. (A) Tibia length of mice at different ages. Multiple tests were performed as general linear hypothesis tests and *P*-values were adjusted for multiple testing using the "single-step" method. Main effects of the two predictors were tested by F-tests in corresponding linear models without interaction terms. n=6-12 mice per group. The sample size for each group is indicated in the Supplementary Table 2. *p <0.05, **p≤0.01, ***p ≤0.001. (B) A retrospective survival analysis was conducted on a cohort of WT (n=18) and *p66Shc* $^{-/-}$ (n=20) mice, which were housed in the animal facility until they reached 24 months of age. Kaplan-Meier curve and Log-rank (Mantel-Cox) test were performed with GraphPad Prizm 8.4 (GraphPad software, USA).

© 2023. Garcia Castro CF et al. Published online at http://www.aginganddisease.org/EN/10.14336/AD.2023.0715

SUPPLEMENTARY DATA

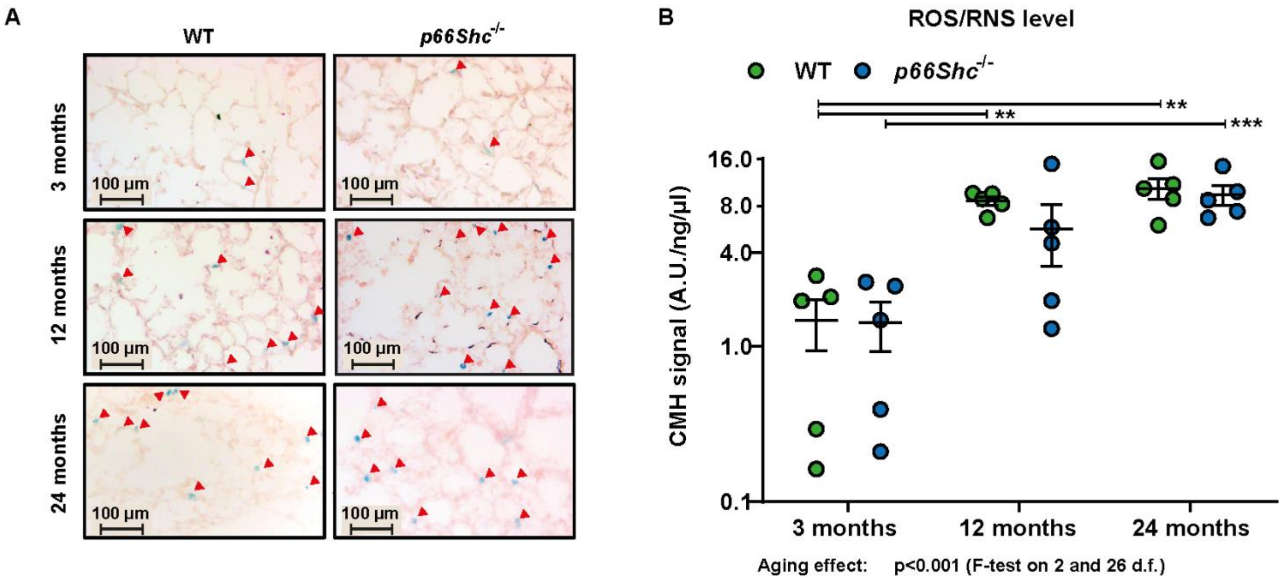

Supplemental Figure 3. The effect of *p66Shc* $^{-/-}$ on β-galactosidase-positive cells and ROS/RNS level in the lung. (A) Representative images of β-galactosidase activity staining in fresh lung tissues were obtained with counterstaining using Nuclear Fast Red. The β-galactosidase-positive cells, highlighted by blue color, are indicated by red arrows. (B) The level of ROS/RNS in lung homogenate at different ages of WT and *p66Shc*$^{-/-}$ mice measured by ESR microscopy using the CMH spin probe. n=5 mice per group. Data were log-transformed for analysis. Multiple tests were performed as general linear hypothesis tests and *P*-values were adjusted for multiple testing using the "single-step" method. If there is no relevant interaction: main effects of the two predictors were tested by F-tests in corresponding linear models without interaction terms. The sample size for each group is indicated in the Supplementary Table 2. **p <0.05,* ***p*≤0.01*,* ****p* ≤0.001.

Supplementary Table 1. Characteristics of lung samples from donor.

Number	Sex	Age (years)	Group
1	M	22	Young age
2	M	30	
3	F	21	
4	M	32	
5	M	40	Middle age
6	M	48	
7	F	46	
8	M	49	
9	M	47	
10	F	72	Old age
11	F	74	
12	M	60	
13	F	62	
14	F	76	

Supplementary Table 2. Sample size of individual groups.

© 2023. Garcia Castro CF et al. Published online at http://www.aginganddisease.org/EN/10.14336/AD.2023.0715

SUPPLEMENTARY DATA

Figure 1

	1a		1b	
	WT	*p66Shc*$^{-/-}$	WT	*p66Shc*$^{-/-}$
**3m**	10	10	10	10
**12m**	11	6	11	6
**24m**	9	8	9	8

	1c		1d	
	WT	*p66Shc*$^{-/-}$	WT	*p66Shc*$^{-/-}$
**3m**	10	9	10	9
**12m**	10	6	11	6
**24m**	9	8	9	8

Figure 2

	2a		2b	
	WT	*p66Shc*$^{-/-}$	WT	*p66Shc*$^{-/-}$
**3m**	7	8	7	8
**12m**	12	6	12	6
**24m**	8	8	8	8

	2c	
	WT	*p66Shc*$^{-/-}$
**3m**	7	8
**12m**	12	6
**24m**	8	8

	2d		2e	
	WT	*p66Shc*$^{-/-}$	WT	*p66Shc*$^{-/-}$
**3m**	5	5	5	5
**12m**	5	5	5	5
**24m**	5	5	5	5

	2f		2h	
	WT	*p66Shc*$^{-/-}$	WT	*p66Shc*$^{-/-}$
**3m**	5	5	10	10
**12m**	5	5	12	6
**24m**	5	5	9	8

Figure 3

3a		3b		3c	
**3m**	**4**	**3m**	**4**	**WT**	6
**12m**	5	**12m**	5	***p66Shc*$^{-/-}$**	6
**24m**	5	**24m**	5		

3d		3e	
**WT**	6	**WT**	6
***p66Shc*$^{-/-}$**	6	***p66Shc*$^{-/-}$**	6

© 2023. Garcia Castro CF et al. Published online at http://www.aginganddisease.org/EN/10.14336/AD.2023.0715

SUPPLEMENTARY DATA

3f		3g		3h	
**WT**	5	**WT**	4	**WT**	5
p66Shc$^{-/-}$	5	***p66Shc$^{-/-}$***	5	***p66Shc$^{-/-}$***	5

	3i		3j	
	**WT**	***p66Shc$^{-/-}$***	**WT**	***p66Shc$^{-/-}$***
**3m**	4	3	3	3
**12m**	4	4	3	3
**24m**	4	4	3	3

	3k		3l	
	**WT**	***p66Shc$^{-/-}$***	**WT**	***p66Shc$^{-/-}$***
**3m**	3	3	3	3
**12m**	3	3	3	3
**24m**	3	3	3	3

Figure 4

	4a		4b	
	**WT**	***p66Shc$^{-/-}$***	**WT**	***p66Shc$^{-/-}$***
**3m**	8	10	9	10
**12m**	11	6	9	5
**24m**	9	8	6	6

	4c	
	**WT**	***p66Shc$^{-/-}$***
**3m**	6	6
**12m**	6	6
**24m**	6	6

Figure 5

	5a		5b	
	**WT**	***p66Shc$^{-/-}$***	**WT**	***p66Shc$^{-/-}$***
**3m**	10	10	10	10
**12m**	12	6	11	6
**24m**	9	8	9	8

	5c		5d	
	**WT**	***p66Shc$^{-/-}$***	**WT**	***p66Shc$^{-/-}$***
**3m**	10	10	10	10
**12m**	12	6	11	6
**24m**	9	7	8	8

© 2023. Garcia Castro CF et al. Published online at http://www.aginganddisease.org/EN/10.14336/AD.2023.0715

SUPPLEMENTARY DATA

	5e		5f	
	WT	*p66Shc*$^{-/-}$	WT	*p66Shc*$^{-/-}$
**3m**	10	10	10	10
**12m**	12	6	12	6
**24m**	9	8	9	8

	5g		5h	
	WT	*p66Shc*$^{-/-}$	WT	*p66Shc*$^{-/-}$
**3m**	10	10	9	7
**12m**	12	6	5	5
**24m**	9	8	7	7

	5i		5j	
	WT	*p66Shc*$^{-/-}$	WT	*p66Shc*$^{-/-}$
**3m**	10	10	10	10
**12m**	12	6	12	6
**24m**	9	8	8	8

Supplement Figure 1

S1a		S1b	
**AECII**	**3**	**3m**	**4**
**mFb**	3	**12m**	5
**PASMC**	3	**24m**	5
**EC**	3		

Supplement Figure 2

	S2a		S2b	
	WT	*p66Shc*$^{-/-}$	**WT**	18
**3m**	10	10	***p66Shc*$^{-/-}$**	20
**12m**	12	6		
**24m**	9	8		

Supplement Figure 3

	S3b	
	WT	*p66Shc*$^{-/-}$
**3m**	5	5
**12m**	5	5
**24m**	5	5

Due to animal availability during experiments, the number of animals in the group varied. In the WT groups, numbers of animals were as follows: n=10, n=12 and n=9 at the age of 3, 12 and 24 months, respectively. In the *p66Shc*$^{-/-}$ groups, numbers of animals were as follows: n=10, n=6 and n=8 at the age of 3, 12 and 24 months, respectively. All animals were used for determination of *in vivo* hemodynamic, echocardiography, lung function measurements and µCT imaging. Randomly assigned mice were chosen for stereology (n=5) and for histological analyses of pulmonary vasculature (n=6). N-numbers for *in vivo* lung function, hemodynamics, echocardiography and µCT may differ from initial n-numbers due to technical issues during measurement (e.g. dislocation of measurement catheter or position of microchip that was used to track mice). The *in vivo* lung function data (stat. compliance, inspiratory capacity, hysteresis and tissue elastance) with coefficient of determination (COD) greater than 0.95, were excluded from analysis.

© 2023. Garcia Castro CF et al. Published online at http://www.aginganddisease.org/EN/10.14336/AD.2023.0715
